# Curing cryptoglandular anal fistulas—Is it possible without surgery?

**DOI:** 10.1016/j.heliyon.2024.e41297

**Published:** 2024-12-16

**Authors:** Chuang Wu, Zubing Mei, Zhenyi Wang

**Affiliations:** a Shanghai Baoshan District Hospital of Integrated Traditional Chinese and Western Medicine, Shanghai, 201999, China; b Shuguang Hospital, Shanghai University of Traditional Chinese Medicine, Shanghai, 201203, China; c Yueyang Hospital of Integrated Traditional Chinese and Western Medicine, Shanghai University of Traditional Chinese Medicine, Shanghai, 200437, China

**Keywords:** Cryptoglandular anal fistula, Anaerobic bacteria, Inflammation products, Nonsurgical treatment

## Abstract

**Background:**

Empirical reviews suggested that cryptoglandular anal fistulas require surgical resolution. However, some reports have indicated the possibility of nonsurgical and conservative treatment, which is discussed in this review.

**Methods:**

This review explores the potential of nonsurgical approaches for curing anal fistulas through bacterial inhibition and immunomodulation. The longstanding cryptoglandular theory has been a subject of controversy, prompting the reevaluation of conventional surgical interventions for anal fistulas. The review was conducted through database searches, including Medline, EMBASE, PubMed, and the Cochrane Library.

**Results:**

Emerging evidence suggests that targeting the anaerobic environments present in anal fistulas and perianal abscesses and eradicating bacteria and their by-products may be critical for successful treatment. Immunomodulatory strategies show promise as a potential avenue for the nonsurgical management of anal fistulas.

**Conclusions:**

Ongoing developments in pharmacological research offer opportunities for alternative treatment options, shedding light on the prospects of noninvasive anal fistula management.

## 1 Introduction

The widespread utilization of surgical treatment for anal fistulas primarily stems from the cryptoglandular theory, wherein management of the internal fistula opening is deemed the gold standard for attaining a cure [1]. This theory, proposed by Parks, asserts that anal crypt infections obstruct anal glands, triggering intersphincteric abscess formation [2]. Abscesses can spread through the longitudinal muscle, yielding various fistulas characterized by their relation to the internal and external sphincters. However, emerging evidence reveals that only a minor proportion of abscesses or fistulas directly communicate with the anal glands [3]. Moreover, the mere closure of the internal opening of the crypt seems inadequate for spontaneous resolution of the infection. The ongoing influx of fecal matter and bacteria from the rectum into the fistulous tract exacerbates the issue. Additionally, the impact of resection is less pronounced than that for abscesses occurring in other organs owing to the anatomical proximity of the anus to the body surface. Consequently, surgical intervention is favored in such cases.

Surgical interventions for anal fistulas, grounded in the cryptoglandular theory, involve the incision of the internal opening and the excision of the fistula tract [4]. Conventional procedures, such as fistulectomy, fistulotomy, and the seton technique, involve excision of the internal opening and fistula tract to achieve healing. However, incomplete excision of the tract or the presence of remnants leads to low cure rates and high recurrence rates of 8–40 % [5]. Conversely, sphincter-sparing procedures, such as ligation of the intersphincteric fistula tract [6], transanal opening of intersphincteric space procedure [7], and anorectal advancement flap [8], primarily address the internal opening without excising the fistula tract and still achieve anal fistula healing. This observation suggests potential limitations in employing the cryptoglandular theory to guide anal fistula treatment. Furthermore, following perianal abscess drainage, local tissue fibrosis develops into an isolated fistula tract disconnected from the external environment. The presence of fibrotic tissue within the fistula significantly hinders fistula closure, unlike recovery from inflammatory and fibrotic conditions in other organs through conservative treatment.

Therefore, this review focuses on exploring the feasibility of curing anal fistulas conservatively by searching Medline, EMBASE, PubMed, and the Cochrane Library for relevant studies on conservative treatment of cryptoglandular anal fistula from 2000 to 2024. Therefore, this review focuses on the feasibility of curing anal fistulas conservatively. With the advancement of minimally invasive surgical techniques, excising the fistula has been increasingly avoided. Consequently, developments in technology and medicine may present opportunities for curing anal fistulas without excising any tissue.

## 2 Challenges to the cryptoglandular theory

In 1880, Herrmann and Desfosses proposed that anal fistulas may arise from anal glands that penetrate the perianal tissue from the rectal submucosa [9]. This hypothesis, now commonly known as the crypt theory, was supported by the findings of Sir Parks in 1961 [10]. He examined 30 fistula specimens, of which eight were associated with anal glands, 13 had transitional epithelium similar to that of the anal canal at the dentate line, and seven contained anal glands that were not part of the fistula tract. Based on these findings, Parks concluded that 90 % of anal fistulas are caused by infected anal glands. However, the sample size in Parks' study was small, and magnetic resonance imaging was not available then. Therefore, the relationship between perianal abscesses or fistulas and perianal tissues remains elusive.

The cryptoglandular theory of anal fistulas has not garnered universal support and has been the subject of mounting criticism in recent years. Many researchers have proposed alternative explanations, including the central intersphincteric space infection and epithelial cell theories. Gottesman argued that the crypt theory is not universally applicable. It can explain how infections that occur within the anal canal, near the internal anal sphincter, can drain inward to form anal fistulas. However, it cannot explain the development of anal fistulas from infections in the intersphincteric space or longitudinal muscle [11]. Histological analysis of 44 specimens of fistulas external to the external anal sphincter and nine specimens of fistulas from the intersphincteric area did not reveal the presence of anal gland tissue in the examined specimens [12]. Based on their analysis of 440 patients with anal fistulas who underwent magnetic resonance imaging, Garg et al. reported that Parks' classification, which is based on the crypt theory, does not accurately reflect the severity of anal fistulas and may lead to incorrect surgical decision-making [13]. Toyonaga et al. found that abscesses associated with anal fistulas were more likely to harbor intestinal-derived microbes rather than skin-derived microbes [14]. Some evidence suggests that intestinal microbiota may facilitate the development of anal fistulas [15,16]; however, other studies demonstrate that the bacterial composition and load in anal fistulas are not significantly associated with chronic inflammation of the fistula tract [17]. These findings suggest that although intestinal bacteria may play a role in certain anal and perianal infections and abscess formation, these bacteria may not be the decisive factor for the persistence of anal fistulas [18].

## 3 Targeting anaerobic environments to improve anal fistula cure rates

Bacteria that are commonly isolated from perianal abscesses, such as *Escherichia coli*, *Bacteroides fragilis*, *Staphylococcus aureus*, and *Streptococcus viridans* [19], typically originate from the colon and do not induce perianal lesions in most instances. However, under anaerobic conditions due to anal gland infections, a chain of reactions ensues, leading to abscess development and subsequent fistula formation.

The use of antibiotics alone is insufficient for treating perianal abscesses and provides limited assistance in addressing anal fistulas [20]. Moreover, antibiotic therapy does not prevent the occurrence of fistulas [21]. However, in another study, metronidazole and ciprofloxacin significantly reduced the rate of fistulization of simple perianal abscesses after incision and drainage [22]. According to a meta-analysis, the use of antibiotics after incision and drainage of anorectal abscesses is correlated with a 36 % lower probability of fistula development [23].

Another recent meta-analysis indicated that the rate of fistula development is substantially lower following perianal abscess incision and seton drainage than after simple incision and drainage. This evidence suggests that maintaining continuous drainage facilitated by a suture or rubber band, which allows air to enter the abscess cavity, supports fistula healing [24]. Further research has demonstrated that negative pressure irrigation applied after perianal abscess drainage significantly reduces the rate of fistula formation [25,26]. Employing multiple incisions to expose all abscess cavities can reduce the occurrence of postoperative fistula to 13 % [27], implying that more extensive exposure of perianal abscesses enhances the probability of spontaneous healing. Nevertheless, the intricate interplay between bacteria and tissues within abscesses and fistulas remains largely unexplored and warrants further investigation.

## 4 Eradicating bacteria and their by-products as a potential approach for nonsurgical anal fistula treatment

Approximately 40 % of perianal abscesses progress to anal fistulas [23]. Clinical observations suggest that inflammatory marker levels in the blood rapidly decrease after draining perianal abscesses, rendering antibiotic use potentially redundant. As inflammation subsides, granulation tissues proliferate, and tissues gradually repair themselves. Despite the periodic manifestation of inflammatory symptoms or fibrosis within the abscess cavity wall, fistulas may not close. This is considered to be due to the continuous ingress of bacteria into the fistula through the internal opening of the crypts, which prevents self-healing.

Nonetheless, research has demonstrated that bacteria do not significantly colonize the fistula wall and that inflammation is absent in this area [26]. Furthermore, studies have reported the persistence of bacterial by-products, such as peptidoglycan and lipopolysaccharide, within fistula tissues. These substances act as immune enhancers that stimulate monocytes and endothelial cells to generate proinflammatory cytokines, including interleukins (IL-1β, IL-6, IL-8, IL-12, and IL-17), tumor necrosis factor-alpha, and interferon [[28], [29], [30]]. However, the antimicrobial peptides generated within anal fistulas act as a defense mechanism, preventing microbes from fecal matter from inducing local or systemic infections via the fistula tract [31]. The epithelial-mesenchymal transition mechanism has also been implicated in the failure of fistulas (or Crohn's disease-associated fistulas) to self-heal [32]. The abnormal extracellular matrix resulting from the epithelial-mesenchymal transition instigates the loss of E-cadherin and increases the expression of protein kinase RNA-like endoplasmic reticulum kinase and nuclear factor kappa B in fistula tissues [29]. Autologous adipose stem cells facilitate anal fistula healing by increasing IL-10 production and promoting regulatory T cell proliferation while suppressing T lymphocytes, thus curbing inflammation and promoting tissue repair [33].

These findings indicate that the persistence of anal fistulas may not be solely due to bacterial entry and inflammation but rather a series of inflammatory responses induced by bacterial derivatives present within the fistula.

## 5 Immunomodulation as a potential approach for nonsurgical anal fistula treatment

Research exploring conservative treatments for anal fistulas remains limited. This article reviews 19 studies on the conservative treatment of anal fistulas conducted between 2000 and 2024, as presented in Table 1. In 21 studies on conservative treatments for anal fistula, a variety of non-surgical interventions were employed. These methods included one study using continuous drainage as a physical therapy, two studies with local application or systemic administration of antibiotics (such as amoxicillin–clavulanic acid), and 18 studies using injection therapies. The injected substances included Permacol™ paste, Argentum-Quartz Solution, adipose-derived stem cells, platelet-rich plasma (PRP), silver nitrate solution (SNS), antibiotics, platelet-rich fibrin (PRF), human amniotic membrane (HAM), and autologous adipose tissue. The number of cases in each study ranged from 1 to 186, with one study serving as a pilot. Follow-up durations varied from 3 to 37 months, and healing rates ranged from 44.4 % to 100 %. While outcomes were inconsistent across studies with smaller sample sizes, larger studies demonstrated a degree of efficacy in conservative treatments. However, conservative approaches have demonstrated potential in managing perianal abscesses among infants and toddlers [34]. In pediatric patients, there is an optimistic perspective that anal fistulas may continue to drain and heal spontaneously following abscess drainage [35], possibly due to the more rapid tissue repair compared with that in adults.

**Table 1:** Summary of studies with conservative treating of anal fistula.

Year	Author	Study type	Intervention	Anatomical classification	*n*	Cure rate %(*n*)	Recurrence%(*n*)	Follow-up(monthes)
2000	Rosen NG et al. [35]	Prospective study	Continuous Drainage Following Incision and Fistulotomy for PA	NA	14	100 %(1)	0	37m(2–99m)
2009	Milito G et al. [36]	multi-centre, prospective, observational Study	Permacol™ paste	IAF; TAF	100	53 %(53)	25 %(25)	12m
2015	Giovanni T et al. [37]	Preliminary Study	Argentum-Quartz Solution	IAF; TAF; EAF	3	66.7 %(3)	33.3 %(1)	4m
2018	P. Lobascio et al. [38]	Pilot study	Adipose-derived stem cells (MYSTEM® EVO Technology)	TAF	1	100 %(1)	0	6m
2018	Giordano P et al. [39]	Prospective, observational Study	Permacol™ collagen paste	IAF; TAF	100	56.7 %(55)	40.2 %(39)	12m
2019	M. Maternini et al. [40]	Prospective, single-arm observational clinical study	Non cross-linked equine collagen (Salvecoll-E gel)	LTAF, MTAF, HTAF	70	78.5 %(55)	21.5 %(15)	12m
2019	de la Portilla F et al. [41]	single-center, randomized, double-blindtrial	PRP vs fibrin glue	IAF, TAF, HTAF	32(PRP) vs 24(fibrin glue)	71.8 %(23)vs58.3 %(14)	28.2 %(9)vs41.7 %(10)	12m
2019	Placer-Galán C et al. [42]	Prospective study	1 % SNS	NA	18	44.4 %(8)	44.4 %(8)	12m
2019	Samuk I et al. [43]	Prospective study	i.v. and oral amoxicillin–clavulanic acid	PA in infants	19	52.6 %(10)	28.5 %(4)	22.4m(6–44m)
2021	Cwaliński J et al. [44]	Preliminary and Prospective trial.	Group I: PRP vs Group Ⅱ: PRP with polyurethane foam or negative pressure wound therapy	IAF; TAF; SAF	8 vs 10	50 %(4) vs 70 %(7)	40 %(7) vs30 %(3)	12m
2021	Attaallah W et al. [45]	Prospective study	5 % SNS	Grade 1–5(St James's Hospital radiologic classification criteria)	186	44.1 % (82)	55.9 % (104)	50m
2022	Garg P et al. [46]	Small retrospective case series	Local and oral antibiotics with avoidance of constipation(LOABAC)	Grade I fistulas(no external opening and only intersphincteric component)	6	66.7 % (4)	33.3 % (2)	24m(12–84m)
2021	Pérez Lara FJ et al. [47]	Prospective, observational Study	PRF	STTAF or SAF	70	52.86 % (37)	14.3 % (10)	24m
2022	Grossi U et al. [48]	Observational Study	HAM	TAF	2	100 % (2)	0	6m
2022	Ratto C et al. [49]	Polit study	HAM	NA	1	100%(1)	0	3m
2023	Dalby HR et al. [50]	Prospective cohort study	Autologous Adipose Tissue	TAF; EAF; SAF	77	51 % (39)	38 % (29)	6m
2023	Ram E et al. [51]	prospective single-arm study	Autologous Blood Clot(RD2-Ver.02)	Simple anal fistula CAF(2022 ASCRS guidelines)	49(Cryptoglandular anal fistula:44, Crohn's Disease:9)	69 % (34)	30.6 % (15)	6–12m
2023	Abdollahi A et al. [52]	Pilot study	PRP + platelet-rich fibrin glue	NA	10	60 % (6)	40 % (4)	33.6m(10–84)
2023	Estella Y. Huang et al. [53]	Prospectively study	Autologous Fat	CAF	52	64.4 % (29)	40.4 %(21)	6m
2024	Xu et al. [54]	Prospective, single-center, open-label, non-randomized, interventional clinical trial	Autologous adipose tissue	HTAF	9	66.6 % (6)	33.3 % (3)	12–36m
2024	Dawoud C et al. [55]	Retrospective study	Autologous compound platelet-rich fibrin foam (Obsidian RFT)	TAF; IAF; EAF; Unclear:2 (13.3)	15[Crohn associated fistula: 4 (26.7) Cryptoglandular associated fistula: 11 (73.3)]	53.3 % (8)	46.7 % (7)	median follow-up of 32 months

**Note:** PA=Perianal abscess; PRP=Platelet-rich plasma; SNS=Silver nitrate solution; IAF=Intersphincteric anal fistula; TAF = Transsphincteric anal fistula; LTAF = Low Transphincteric anal fistula; MTAF = Median Transphincteric anal fistula; HTAF=High Transphincteric anal fistula; STTAF=Single-tract transsphincteric anal fistula; SAF=Suprashincteric anal fistula; EAF = Extrasphincteric anal fistula; CAF=Complex Anal Fistulas; BAF=Branched Anal Fistulas; HTAF=High transphincteric anal fistula.

Anal fistulas are not purely the result of an inflammatory response, but are intimately connected with immune abnormalities. Secondary fistulas may be more complex and prone to recurrence, likely due to exacerbated immune abnormalities [56]. Both systemic and local administration of immunosuppressive agents are beneficial in controlling symptoms and treating anal fistulas [57,58]. Anti-tumor necrosis factor-alpha therapy even shows promise as a conservative cure for Crohn's disease-associated fistulas [59]. Garg et al. demonstrated that topical application of antibiotics (povidone-iodine and ornidazole cream) in conjunction with oral antibiotics (ciprofloxacin and ornidazole) and maintenance of regular bowel movements can cure stage I anal fistulas (four out of six cases were successfully treated) [46], demonstrating its potential for conservative treatment.

Moreover, sealing fistulas(and suturing the internal opening) with platelet-rich fibrin gel achieved a 52.86 % cure rate, suggesting its potential as an initial conservative approach to treating anal fistulas [47]. In the study by Dawoud et al., 15 fistula cases were treated with autologous compound platelet-rich fibrin foam (Obsidian RFT) for fistula closure. The median follow-up duration was 32 months, with a healing rate of 53.3 %, and no complications were observed [55]. Xu et al. [54]. conducted a meta-analysis of 19 studies involving PRP treatment in 418 anal fistula patients. The analysis revealed an overall healing rate of 68 % for PRP, with a pooled healing rate 1.35 times that of the control group (pooled OR = 1.35, 95 % CI, 1.14–1.60, P < 0.001). For complex anal fistulas, collagen matrix injections and stromal stem cells have demonstrated impressive efficacy [60,61]. Although stem cell therapy was initially employed primarily for Crohn's disease or Crohn's disease-associated fistulas, its use in the treatment of cryptoglandular anal fistulas is increasing due to their association with immune abnormalities. However, these therapies typically require prior suturing of the internal opening [50].

The principle behind the application of autologous fat grafting for treating anal fistulas stems from the rich presence of various growth factors within autologous fat, including platelet-derived growth factor, fibroblast growth factor, and vascular endothelial growth factor, which can effectively repair the fistula cavity. Additionally, the adipose tissue harbors adipose-derived stem cells with the potential to differentiate into various cell types to heal anal fistulas, with reported cure rates ranging from 48 % to 66.6 % [[42], [62], [63]]. Moreover, autologous fat is readily accessible and easier to procure than stem cells, as it offers an economical alternative. The administration with a 1 % silver nitrate solution has shown a 44 % cure rate for treating anal fistulas (without the need for suturing the internal opening) because of its bacteriostatic and anti-inflammatory properties and its ability to coagulate proteins and exert astringent effects on the fistula [36]. Furthermore, other reports suggest cure rates ranging from 50 % to 73 % [37,38].

There are other reported treatments for anal fistulas, such as the injection of an autologous blood clot (RD2-Ver0.2) after suturing the internal opening, which has demonstrated a 60 % (33 out of 53) cure rate over a 12-month follow-up [51]. Techniques such as stuffing the fistula tract with the human amniotic membrane after debridement and suturing of the internal opening, as reported by Grossi et al., take advantage of its wound-healing, anti-inflammatory, antimicrobial, and anti-fibrotic properties [53]. Although these treatments for anal fistulas have indicated considerable therapeutic effects, they are still at preliminary stages and warrant additional trials for further investigation and verification.

## 6 Conclusions

Considering the perspectives discussed above, we posit that the key to a nonsurgical cure for anal fistulas encompasses two major aspects. First, reducing bacterial presence within the fistula tract is critical, either by preventing intestinal bacteria from entering the opening or rapidly inactivating anaerobic bacteria at the site. Second, addressing bacteria and their derivatives within the fistula tract requires the reversal of immune abnormalities. Consequently, immediate closure of the internal opening may not be the sole solution to cure anal fistulas. Instead, promoting the transition of anaerobic bacteria within the fistula tract into an aerobic state over an extended period, accompanied by pharmaceutical interventions to correct immunological abnormalities and remove inflammatory factors, may be a promising approach toward the pharmacological cure of anal fistulas. However, there are currently no published studies on using anti-TNF-α therapy for non-Crohn's anal fistulas. This may be because the immune dysregulation in cryptoglandular anal fistulas differs from that seen in Crohn's-related fistulas. Although there is currently no literature on this topic, a short-term study could be considered, such as observing the therapeutic effect of locally applying anti-TNF-α agents after suturing the internal opening of the anal fistula.

Nevertheless, reversing tissue immune abnormalities is complex. For example, reversing abnormal immunity in the intestinal mucosa in inflammatory bowel diseases is demanding. However, anal fistulas are considerably less complex than inflammatory bowel diseases. As medical and material technologies advance, a pharmacological cure for anal fistulas may become feasible. At present, it seems prudent to consider trialing drugs used to treat Crohn's disease-associated fistulas for conservative treatment. Nonetheless, this necessitates further research and substantiation.

We contend that investigating the relationship between bacteria infecting anal fistulas and tissue anaerobic states and examining immune abnormalities while repairing fistula tissues may prove critical in unlocking a pharmacological cure for anal fistulas. Utilizing existing drugs or developing new ones aimed at eradicating anal gland infections and addressing fistula fibrous tissues may represent the future direction in nonsurgical anal fistula treatment.

## CRediT authorship contribution statement

**Chuang Wu:** Writing – review & editing, Methodology, Investigation. **Zubing Mei:** Methodology, Investigation. **Zhenyi Wang:** Writing – original draft, Conceptualization.

## Data and code availability statement

All data analyzed in this review are derived from previously published studies, which are cited within the article and listed in the References section.

## Funding

This study was supported by Project No. 2024YJJB09, titled *“**Clinical Efficacy Observation of Lianbai Enema Formula in the Treatment of Active Ulcerative Colitis and Related Transformative Research**.”*

## Declaration of competing interest

The authors declare the following financial interests/personal relationships which may be considered as potential competing interests: The authors declare the following competing interests:Zhenyi Wang is employed by Yueyang Hospital of Integrated Traditional Chinese and Western Medicine, Shanghai University of Traditional Chinese Medicine. All other authors declare no competing interests.
